# 

**Published:** 1908-03

**Authors:** 


					CONTENTS.
Past
?he Long Fox Lecture: Suppurative Disease in the Nose and
Ear.
By Patrick Watson Williams, M.D.Lond.
Typhoid Fever and Typhoid Carriers.
By D. S. Davies, M.D. Lond.,
and I. Walker Hall, M.D.Vict.
39
Operative Interference in Carcinoma of the Pancreas.
By James Swain, M.S., M.D. Lond., F.R.C.S. ...
45
The Treatment of Spreading Peritonitis by Drainage, the Fowler
Position, and Rectal Instillation of Saline Solution, with Notes
of a Second Successful Case.
By C. Hamilton Whiteford
53
Case of Rupture of Middle?Meningeal Artery: Operation;
Reaovery.
By G. Munro Smith
58
Abdominal Fibroma Simulating Splenomegaly.
By David A. Alexander, M.B.Edin.
6o
Progress of the Medical Sciences :
Medicine.
J. R. Charles, M.A., M.D. Camb., M.R.C.P.
6i
Surgery.
E. W. Hey Groves, M.S. Lond., F.R.C.S.
65
Reviews of Books
6q
Editorial Notes
83
Notes on Preparations for the Sick
86
The Library of the Bristol Medico-Chirurgical Society   89
Meetings of Societies
92
Local Medical Notes
94

				

